# Somatic Copy Number Alterations in Circulating Cell-Free DNA as a Prognostic Biomarker for Hepatocellular Carcinoma: Insights from a Proof-of-Concept Study

**DOI:** 10.3390/cancers17071115

**Published:** 2025-03-26

**Authors:** Elisa Pinto, Elisabetta Lazzarini, Filippo Pelizzaro, Martina Gambato, Laura Santarelli, Sara Potente, Paola Zanaga, Teresa Zappitelli, Romilda Cardin, Patrizia Burra, Fabio Farinati, Chiara Romualdi, Diego Boscarino, Valeria Tosello, Stefano Indraccolo, Francesco Paolo Russo

**Affiliations:** 1 Department of Surgery, Oncology and Gastroenterology, University of Padova, 35121 Padua, Italy; pintoelisa93@gmail.com (E.P.); filippo.pelizzaro@gmail.com (F.P.); martina.gambato@gmail.com (M.G.); paola.zanaga@unipd.it (P.Z.); teresa.zappitelli@unipd.it (T.Z.); burra@unipd.it (P.B.); fabio.farinati@unipd.it (F.F.); stefano.indraccolo@unipd.it (S.I.); 2 Gastroenterology Unit, Azienda Ospedale-Università di Padova, 35121 Padua, Italy; romilda.cardin@unipd.it; 3 Basic and Translational Oncology Unit, Veneto Institute of Oncology IOV-IRCCS, 35121 Padua, Italy; elisabetta.lazzarini@iov.veneto.it (E.L.); laura.santarelli@iov.veneto.it (L.S.); valeria.tosello@iov.veneto.it (V.T.); 4 Department of Biology, University of Padova, 35121 Padua, Italy; sara.potente@phd.unipd.it (S.P.); chiara.romualdi@unipd.it (C.R.); 5 AB Analitica, 35127 Padua, Italy; boscarino@abanalitica.it

**Keywords:** HCC, liquid biopsy, sWGS, ctDNA

## Abstract

Background/Objectives: Despite advances in hepatocellular carcinoma (HCC) management, prognosis remains poor. Advanced-stage diagnosis often excludes curative treatments, and current biomarkers (e.g., alpha-fetoprotein [AFP]) have limited utility in early detection. Liquid biopsy has emerged as a promising cancer detection tool, with circulating cell-free DNA (ccfDNA) showing significant diagnostic potential. This proof-of-concept study aimed to investigate the potential role of tumor fraction (TF) within ccfDNA as a biomarker in HCC patients. Methods: A total of sixty patients were recruited, including thirteen with chronic liver disease (CLD), twenty-four with cirrhosis, and twenty-three with HCC. Plasma samples were collected, and ccfDNA was extracted for shallow whole genome sequencing (sWGS) analysis. The TF was calculated by focusing on somatic copy number alterations (SCNAs) within the ccfDNA. Results: Among patients with CLD and cirrhosis (n = 37), ctDNA was undetectable in all but one cirrhotic patient who exhibited a significant tumor fraction (TF) of 17% and subsequently developed HCC. Conversely, five out of twenty-three HCC patients (21.7%) displayed detectable ctDNA with TF levels ranging from 3.0% to 32.6%. Patients with detectable ctDNA were characterized by more aggressive oncological features, including a higher number of nodules (*p* = 0.005), advanced-stage disease (60% BCLC C, *p* = 0.010), and poorer response to therapy (80% PD, *p* = 0.001). Moreover, the overall survival (OS) was significantly reduced in patients with detectable ctDNA (median OS: 17 months; CI 95% 4.5–26.5) compared to those without (median OS: 24.0 months; CI 95% 7.0–66.0; log-rank *p* = 0.002). Conclusions: Our results suggest that the analysis of TF by sWGS is a promising non-invasive tool for the identification of HCC with aggressive clinical behavior, whereas it is not sensitive enough for early HCC detection. This molecular assay can improve prognostic stratification in HCC patients.

## Simple Summary

Hepatocellular carcinoma (HCC) continues to pose challenges due to obstacles in early diagnosis, predicting treatment response, and accurate prognostic evaluation. Current biomarkers, such as alpha-fetoprotein (AFP), exhibit limited sensitivity. This study investigates the role of tumor fraction (TF) in circulating cell-free DNA (ccfDNA) as a potential biomarker for HCC. Plasma samples from sixty patients with chronic liver disease, cirrhosis, and HCC were analyzed using shallow whole genome sequencing (sWGS). Detectable circulating tumor DNA (ctDNA) was identified in 21.7% of HCC patients and correlated with more aggressive tumor characteristics, poorer treatment response, and reduced overall survival. Although the TF analysis lacked sufficient sensitivity for early detection, it was valuable for identifying high-risk patients and refining prognostic stratification. These findings suggest that TF measurement via sWGS could serve as a non-invasive tool to enhance prognosis assessment and guide personalized treatment strategies in HCC management.

## 1. Introduction

Hepatocellular carcinoma (HCC) is the sixth most commonly diagnosed cancer and the third leading cause of cancer-related deaths globally [1]. Despite significant improvements in disease management, the prognosis for patients with HCC remains poor, with a 5-year survival rate of nearly 20% [2]. This poor outcome may be partly due to a late diagnosis, which often precludes curative treatments despite the availability of surveillance strategies. Reliable biomarkers for early diagnosis and accurate prognostic stratification are currently lacking, highlighting a critical gap in HCC research [3]. Indeed, alpha-fetoprotein (AFP), the universally recognized diagnostic and prognostic biomarker in HCC [4], has several limitations [5,6,7,8]. As a result, considerable efforts have been made to identify biomarkers for early detection, prognostic evaluation, and predictive stratification. Recently, several studies have demonstrated that AFP-L3, the Lens culinaris agglutinin-binding fraction of AFP, and des-γ-carboxy prothrombin (DCP) are specific biomarkers for HCC [9]. Moreover, miRNAs, such as miR-21, miR-122, and miR-221, may offer better sensitivity than traditional markers like AFP in certain settings [10].

In this context, liquid biopsy (LB) is an emerging non-invasive technique for tracking various tumor-derived circulating analytes [11,12,13,14,15], including circulating cell-free DNA (ccfDNA), which is considered one of the most promising biomarkers for HCC [15].

Somatic copy number alterations (SCNAs), tumor-specific copy number variations (CNVs), can be detected in peripheral blood, allowing for the inference of tumor fraction (TF) in plasma [16]. TF represents the proportion of circulating tumor DNA (ctDNA) within the total ccfDNA in plasma and has been shown to correlate with tumor burden and cancer prognosis [17,18,19,20]. TF can be inferred through shallow whole genome sequencing (sWGS) of ccfDNA [21], offering a cost-effective approach for clinical applications [22,23].

This proof-of-concept study aimed to analyze TF in patients with chronic liver disease (CLD), cirrhosis, and HCC to gain a deeper understanding of its clinical significance across these distinct conditions.

## 2. Materials and Methods

### 2.1. Patients

A total of sixty patients between October 2021 and March 2024 were prospectively enrolled. This study included thirteen patients with chronic CLD, twenty-four patients with cirrhosis, and twenty-three patients with HCC. Each participant provided written informed consent to participate in the study, which was conducted in compliance with the Declaration of Helsinki and received approval from the Ethics Committee of Padova University Hospital (approval n. 46093).

Patients with chronic liver disease due to hepatitis B virus (HBV), HBV/hepatitis D virus (HDV), or hepatitis C virus (HCV) infection were enrolled during their routine follow-up. A hepatology specialist diagnosed cirrhosis based on a combination of clinical assessments, laboratory results, imaging, and elastography, detecting stiffness > 12.5 kPa. For HCC patients, we included both those who received previous treatment and those treatment-näive. The presence of HCC was defined according to guidelines available at the time of the diagnosis [24,25].

The following clinical and tumor-related parameters were recorded in each group of patients: sex, age, etiology of the underlying liver disease, and key serological parameters [total bilirubin, INR, creatinine, albumin, and AFP (ELISA technique; ECLIA Elettrochemiluminescenza. Cobas 602 Roche. CV%: 1.8)]. Additionally, tumor characteristics, such as the number and size of liver nodules, were documented.

### 2.2. Plasma Collection and ccfDNA Extraction

Approximately 20 mL of peripheral blood was collected from each patient into two Cell-Free DNA BCT tubes (Streck Corporate, La Vista, NE, USA) and processed within 24 to 72 h. The samples underwent an initial centrifugation at 2000× *g* for 10 min at +4 °C, followed by a second centrifugation of the plasma at 20,000× *g* for an additional 10 min at room temperature. The resulting supernatant was stored at −80 °C until ccfDNA extraction.

ccfDNA was extracted from 2 to 5 mL of plasma using the Maxwell^®^ RSC ccfDNA LV Plasma Kit (Promega, Milan, Italy), following the manufacturer’s protocol. The extracted ccfDNA was quantified using the Qubit dsDNA HS assay kit on a Qubit 3.0 fluorometer (Thermo Fisher Scientific, Waltham, MA, USA).

### 2.3. Shallow Whole Genome Sequencing and Bioinformatic Analysis

Whole genome libraries were prepared from 10 to 50 ng of ccfDNA using the KAPA HyperPrep Kit (Roche, Monza, Italy), following the manufacturer’s instructions. Sequencing was performed on the Illumina NextSeq 500/550 platform (Illumina Inc, San Diego, CA, USA) with 75 single-end reads, achieving an average genome-wide fold coverage of 0.3× to 0.7×.

Both segmental copy number variations (CNVs) and tumor fraction (TF) were inferred using SAMURAI [24] and ichorCNA algorithm [20], which has been reported to detect tumor presence with a sensitivity of 0.95 and a specificity of 0.91, using a TF cutoff of 0.03.

### 2.4. Statistical Analysis

Patients were categorized into two groups based on the detectability of ctDNA. Categorical variables are presented as absolute and relative frequency (percentage), while continuous data are expressed as median and interquartile range (IQR). Differences between groups were evaluated using a chi-square test and Fischer exact test for categorical variables and a Mann–Whitney test for continuous variables, as appropriate. Survival curves analyses were performed using the Kaplan–Meier method, with comparisons between groups conducted via the log-rank test. The p value (two-tail) was considered statistically significant when <0.05. IBM SPSS Statistics (Version 25.0. IBM Corp, Armonk, NY, USA) was used for statistical analyses.

## 3. Results

### 3.1. Patient Characteristics

Baseline characteristics of the study population are summarized in Table 1.

Across all three patient groups, the majority were male (53.8% vs. 58.3% vs. 73.9%), with a median age of over 50 years [54.0 (26–70) vs. 60.7 (44–86) vs. 66.7 (45–84)]. The etiology of the CLD control group was exclusively viral, whereas the cirrhosis and HCC groups exhibited diverse etiologies. Liver function was largely preserved in both the cirrhosis and HCC groups (MELD: 12 vs. 10; CHILD A: 75.0% vs. 82.6%). Additionally, the majority of patients in both groups showed clinically significant portal hypertension (CSPH) (66.7% vs. 73.9%).

Regarding AFP levels, the values varied between the three groups, being higher in the HCC group [2.1 (0.7–4.6) vs. 6.3 (2.0–38.1) vs. 20176.0 (1.5–22,700.0)]. During a median follow-up of 71.7 months (2.0–1493.0) for the cirrhotic group, four patients were diagnosed with HCC.

Not all patients in the HCC group had active nodules, as 39.1% had been previously treated successfully according to mRECIST [complete response (CR), partial response (PR), stable disease (SB), progression disease (PD)]. HCC patients had a variable number of nodules [2.9 (0–15)] of heterogeneous size [31.8 (12.0–110.0)]. Only one patient presented with lung metastasis, resulting in patients being distributed across different BCLC stages depending on liver function and performance status [0: 2 (8.7%) vs. A: 10 (43.8%) vs. B: 4 (17.4%) vs. C: 3 (13.0%)].

During the follow-up period, which had a median duration of 388.7 months (4.0–1428.0), none of the patients with CLD died, while four patients in the cirrhotic group and three in the HCC passed away.

### 3.2. ctDNA Evaluation

In plasma samples collected from patients with CLD, cirrhosis, and HCC, ccfDNA concentrations differed significantly among the groups (*p* = 0.017). The mean ccfDNA concentrations were as follows: 3.51 ± 1.48 ng/mL (median: 2.91 ng/mL; range: 2.29–7.46 ng/mL) for CLD, 10.80 ± 6.08 ng/mL (median: 9.84 ng/mL; range: 3.99–28.80 ng/mL) for cirrhotics, and 14.43 ± 15.92 ng/mL (median: 8.07 ng/mL; range: 2.83–62.67 ng/mL) for HCC patients (Figure S1).

#### 3.2.1. CLD and Cirrhosis

**Figure 1:**
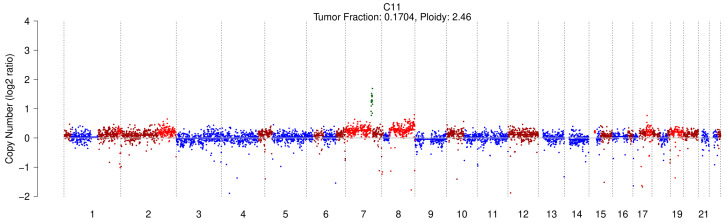
sWGS profile and TF of the cirrhotic patient with detectable ctDNA.

None of the patients with CLD exhibited alterations in their circulating genomic profile, with ctDNA levels consistently undetectable (TF = 0 for all CLD samples) (Table S1; Figure S2). However, one cirrhotic patient displayed an altered profile, particularly with a TF of 17.0% (Figure 1). This patient was a 69-year-old male with decompensated alcoholic cirrhosis (CHILD B9, MELD 12) and signs of portal hypertension such as ascites and esophageal varices. At the time of sampling, a computed tomography (CT) scan revealed regenerative nodules with uncertain characteristics, which remained unclassified as HCC even after an MRI was performed 2 days later. However, after two months, a repeated CT scan identified these nodules as consistent with HCC, suggesting that the patient likely had undiagnosed HCC at the initial sample collection. Notably, AFP levels remained within the normal range throughout this period. The patient died two months after the sample was collected due to acute decompensation of his cirrhosis.

#### 3.2.2. HCC Patients

**Figure 2:**
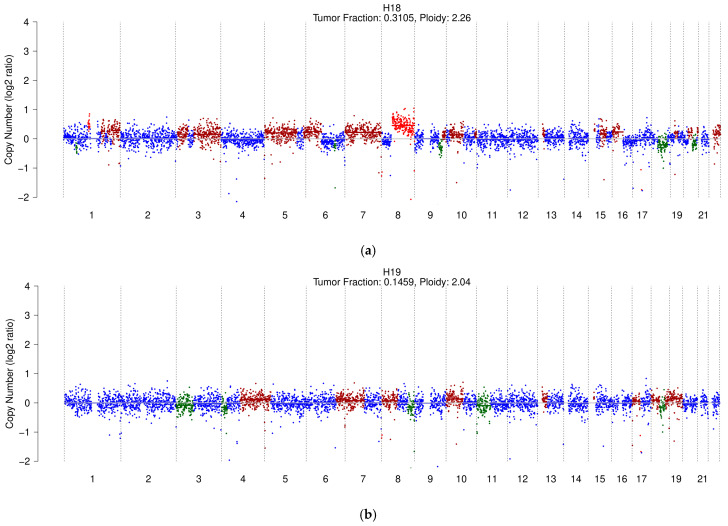

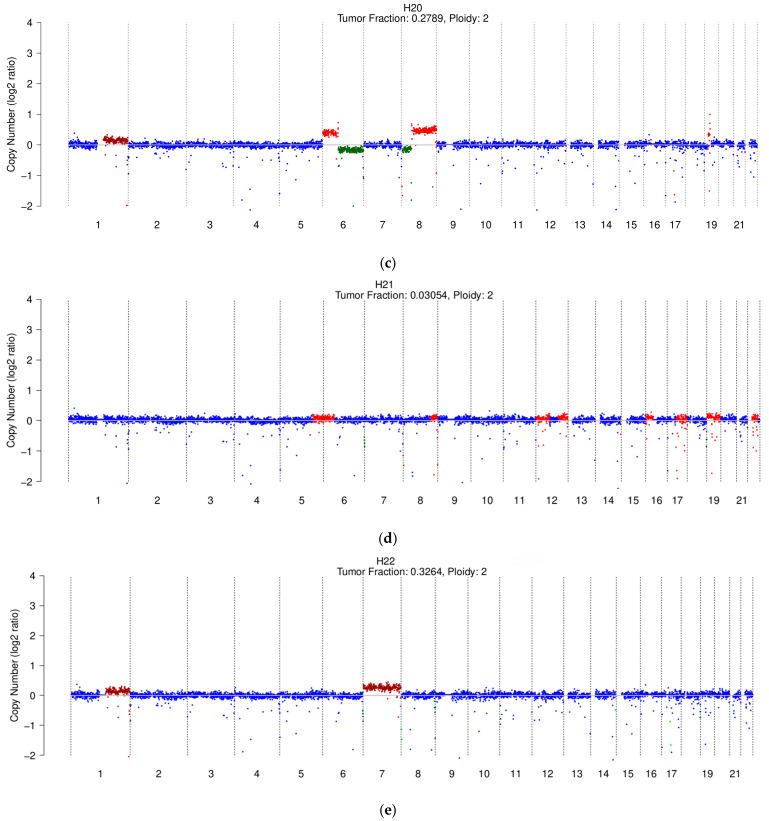
sWGS profiles and TF of HCC patients with an altered profile: (**a**) H18; (**b**) H19; (**c**) H20; (**d**) H21; (**e**) H22. The colors blue, green, and red correspond to copy number neutral, deletion, and amplification, respectively.

Only five out of twenty-three patients with HCC exhibited an altered genomic profile with detectable ctDNA (Figure 2), while ctDNA was undetectable in the remaining eighteen patients (Table S1; Figure S2). As shown in Table 2, these patients had highly heterogeneous AFP levels (range 4.6–22,700.0 ng/mL), and each was diagnosed with more than three nodules, with maximum diameters ranging from 17.0 to 55.0 mm.

The TF percentage ranged from 3.1% to 32.6% (mean 21.84% ± 12.65; median 27.9%). Three patients with the highest TF percentages were classified as BCLC C. Two also had the highest AFP levels, and one had metastasis.

Among all patients with HCC, the three patients who died had all detectable TF, with the patient showing the highest TF levels experiencing the shortest survival (1 month).

Table 3 summarizes the main characteristics of HCC patients without detectable ctDNA compared to those with an altered profile. The two groups presented a similar demographic characteristics distribution with a male sex predominance (66.7% vs. 100%, *p* = 0.133) and a median age > 60 years old. No differences were detected in terms of etiology, with viruses being the leading cause of liver disease in both groups (55.6% vs. 60.0%, *p* = 0.461). In both groups, the liver function was predominantly preserved, with most patients presenting a CHILD Pugh score A (83.3% vs. 80.0%, *p* = 0.109).

Patients presented similar levels of AFP [6.8 (3.2–192.5) vs. 8.6 (3.8–11585), *p* = 0.359) and maximum size of the nodules [24.0 (17.0–46.0) vs. 20.0 (16.0–38.0), *p* = 0.500]. However, patients with detectable ctDNA presented with a higher number of nodules [1 (1–4) vs. 4.7 (1–11), *p* = 0.005], resulting in a higher percentage of BCLC C stage patients (0% vs. 60.0%, *p* = 0.010). In both groups, some patients were previously treated (55.6% vs. 80.0%). Although there were no significant differences in HCC activity (*p* = 0.246), we observed that in the detectable ctDNA group, all the patients presented with active nodules. In contrast, in the undetectable ctDNA group, only 77.8% of patients had active HCC, with the remaining 22.2% successfully treated.

All the patients in the detectable ctDNA group did not respond to treatment, presenting with a higher percentage of PD according to mRECIST (0% vs. 80.0%, *p* = 0.001).

Finally, the overall survival (OS) for the entire HCC study population was a median of 20.5 months (CI 95% 7.0–48.25). When stratified by ctDNA status, patients without detectable ctDNA had a median OS of 24 months (CI 95% 7.0–66.0), whereas those with detectable ctDNA had a significantly shorter median OS of 17 months (CI 95% 4.5–26.5; log-rank *p* = 0.002).

### 3.3. Copy Number Profiles in Cirrhosis and HCC

Figure 2 shows the genomic profiles of the five HCC patients with detectable ctDNA.

In addition to TF estimation, sWGS in our cohort enabled the detection of SCNAs. The main chromosomal alterations identified in five HCC patients included a 1q gain in three out of five patients (H20, H18, H22), a gain of chromosome 7 (H18, H22), an 8q gain (H20, H18), 6p gain (H20, H18), 10p gain (H19, H18), and an 18q deletion (H19, H18).

While most of these SCNAs have previously been described in the literature [23,26], the full chromosome 7 gain and the 18q deletion, instead of the commonly reported 18q gain, are notable exceptions.

Interestingly, the cirrhotic C11 patient shared several of these chromosomal alterations with HCC samples, including the 1q gain, 7 gain, and 10p gain. Additionally, the C11 patient exhibited a gain of chromosome 2, along with other less extended CNVs, suggesting a possible overlap in genomic alterations between cirrhosis and HCC.

As shown in Table 2, patients with 8q amplification had the highest AFP levels and were classified as BCLC C.

## 4. Discussion

Liquid biopsy has emerged as a promising tool for early cancer diagnosis, prognostic evaluation, and monitoring treatment response. Among various techniques, the analysis of ccfDNA is gaining significant attention for its ability to provide comprehensive insights into a patient’s genomic profile. In particular, the quantification and genomic analysis of ctDNA are key components of this approach. This study aims to preliminarily explore the potential utility of ctDNA quantification for early diagnosis and prognostic evaluation in patients with HCC, a malignancy for which, despite considerable efforts, a standardized method to reliably assist clinicians in comprehensive patient assessment has yet to be established in clinical practice.

In our cohort, patients with CLD did not exhibit any genomic alterations, and none of them subsequently developed HCC. However, longer follow-up periods would be necessary to strengthen the apparently negative predictive value of ctDNA quantification for prognostic stratification in these early stages. Conversely, the potential positive predictive value of ctDNA has recently been investigated, yielding promising results [27,28], further emphasizing the need for extended follow-up studies [29,30].

On the other hand, among cirrhotic patients, four developed HCC during follow-up, but only one of these exhibited an altered genomic profile at the time of sampling. This patient likely had existing HCC nodules at the time of sampling, although radiological investigations were inconclusive. In this case, ctDNA detection could have supported HCC diagnosis by helping to differentiate potentially malignant nodules. This finding aligns with some reports in the literature. Indeed, Fateen et al. recently compared patients with low-grade dysplasia to those with high-grade dysplasia to identify genomic differences [31]. They found that using low-pass DNA sequencing, alterations 1q+ and 8q+ were significantly associated with either early cancer or high-grade dysplasia. In contrast, such events were rare in low-grade dysplasia or cirrhosis [31]. Importantly, in the remaining three cirrhotic patients of our cohort who 5–9 months later developed HCC, ctDNA was not detected, thus suggesting that the sensitivity of this assay is still inadequate for the detection of early cancer. Further studies are needed to assess the possible early diagnostic value of TF evaluation in this setting as a supplementary test that could be used in addition to AFP.

It should be noted that previous studies analyzing the role of ccfDNA in early HCC diagnosis have focused on either the total amount of ccfDNA [32,33,34], or the evaluation of predefined gene panels [35], or the study of methylation patterns [36]. More recently, the first study exploring the role of ctDNA quantification (TF) and DNA copy number profiling in the early diagnosis of HCC using samples from patients before diagnosis has been published [28]. Lian et al. reported that in cirrhotic patients without HCC, the mean TF was 1.1% (±0.7%), with a specificity of 97.8% (95% CI: 92.2–99.7%) in detecting HCC. They also observed that the TF in pre-HCC samples progressively increased as HCC diagnosis approached, with sensitivities ranging from 0.0% to 22.7% in the five years preceding the diagnosis [28]. Although these findings are promising, our study did not confirm the sufficient sensitivity of sWGS in this clinical setting, highlighting the need for further research to draw definitive conclusions.

In our HCC cohort, patients with detectable TF exhibited more aggressive tumor characteristics at the time of sample collection, including a higher number of nodules and more frequent BCLC stage C classification. While AFP levels did not differ significantly between the TF+ and TF− groups, metastases were found exclusively in patients with detectable ctDNA. While not observed in our cohort, AFP elevation can also occur in germ cell malignancies [37], and reproductive organ metastases should be considered in larger studies. Additionally, the detectable ctDNA group showed a substantially broader IQR for AFP levels, with notably higher values at the 75th percentile than patients without detectable ctDNA, and the patient with the highest AFP value (22,700.0 ng/mL) belongs to this group. These data align with the literature suggesting a correlation between ccfDNA concentration in plasma and HCC grading [32,38,39,40,41,42,43,44]. Similarly to our results, Lian et al. reported a significant correlation between mean TF and tumor burden, and over 36 months, patients with a TF above 10% had poorer survival, especially in stage C HCC, highlighting TF potential as a prognostic marker in advanced HCC [28]. Additionally, Sogbe et al. [23] showed that in a cohort of seventy-three patients with HCC, patients with detectable ctDNA presented with a significantly higher probability of being classified as BCLC B/C (*p* < 0.0001), having macrovascular invasion (*p* = 0.023), extrahepatic spread (*p* < 0.0001), larger tumor size (*p* < 0.0001), and higher levels of AFP (*p* < 0.0001), confirming the relation between ctDNA and HCC aggressiveness. Collectively, these findings suggest that TF positivity may indicate a pro-metastatic tendency in HCC, consistent with the shorter OS observed in TF-positive patients. This hypothesis warrants further investigation, such as exploring pro-metastatic gene expression signatures in HCC tumor samples. Moreover, confirming these data in patients at intermediate stages could ensure better prognostic stratification, enabling more appropriate therapeutic decisions in favor of locoregional versus systemic therapies.

Another interesting finding concerns the response to therapies. Among our cohort of HCC patients, fourteen had undergone prior treatments, with four achieving CR according to mRECIST and eradicating the neoplastic disease at the time of sampling. None of these four patients had detectable ctDNA. Despite having active nodules, the remaining patients without detectable ctDNA showed a PR (40%) to previous treatments or SD (20%). In contrast, patients with detectable ctDNA who had undergone previous treatment experienced disease progression (PD: 100%), with two of them subsequently dying, resulting in a significantly poorer prognosis. These findings align with existing literature [45,46,47,48,49], highlighting the potential role of ctDNA as a predictive biomarker for therapy outcomes in HCC patients.

Although the findings are promising, it should be noted that most studies focus primarily on total ccfDNA amounts, genomic instability, and gene panels, with only a few addressing CNVs and the quantity of ctDNA [50,51]. In this regard, one of the most notable studies is by Hsu et al. [51], which showed that in patients with unresectable HCC treated with atezolizumab + bevacizumab, 70% of those with complete responses transitioned from having detectable ctDNA to ctDNA negativity. This transition was not observed in any case where the disease was progressing. Moreover, they noted that patients who achieved ctDNA negativity had longer progression-free survival.

Regarding SCNAs, although our sample size is limited and does not permit definitive conclusions, this study introduces a novel methodology for investigating TF in ccfDNA through the detection of SCNAs. Notably, two of the five altered profiles exhibited the same amplification in chromosome 8q, which has been identified in previous studies as significant in HCC patients [52,53]. A Chinese study reported that 33.3% of 66 HCC patients exhibited copy number gains in 8q, which were associated with significantly reduced survival [52]. These findings were recently corroborated by Pan et al., who demonstrated the prognostic importance of copy number variations in 8q24 genes [53].

Chromosome 8q harbors several oncogenes, including Myc, ATAD2, SQLE, PVT1, ASAP1, and NDRG1, which are believed to play critical roles in the carcinogenesis and progression of HCC.

## 5. Conclusions

While our results are promising and consistent with the findings of the only two similar studies available [23,28], the small sample size remains a limitation and prevents us from drawing robust inferences.

In conclusion, our findings underscore the potential utility of TF evaluation in identifying HCC patients with a higher tumor burden, which could enhance prognostic assessments and assist clinicians in the treatment decision-making process as well as in evaluating treatment responses. Further studies with larger cohorts are necessary to validate these encouraging results.

## Figures and Tables

**Table 1:** Baseline features of the patients.

	Chronic Hepatitis (n = 13)	Cirrhosis (n = 24)	HCC (n = 23)
Sex-n (%)			
Male	7 (53.8%)	14 (58.3%)	17 (73.9%)
Female	6 (46.2%)	10 (41.7%)	6 (26.1%)
Age at the time of the sample—median (IQR)	54.0 (26–70)	60.7 (44–86)	66.7 (45–84)
Etiology-n (%)			
Viral	13 (100%)	10 (41.7%)	13 (56.5%)
Alcohol	-	5 (20.8%)	5 (21.7%)
Viral + Alcohol	-	2 (8.3%)	3 (13.0%)
MetALD	-	3 (12.5%)	2 (8.7%)
MASLD	-	3 (12.5%)	-
Other	-	1 (4.2%)	-
MELD—median (IQR)	-	12 (7–20)	10 (7–24)
CHILD-n (%)	-		
A		18 (75.0%)	19 (82.6%)
B		4 (16.7%)	3 (13.0%)
C		2 (8.3%)	1 (4.4%)
CSPH-n (%)	-		
Yes		16 (66.7%)	17 (73.9%)
No		8 (33.3%)	6 (26.1%)
AFP (ng/mL)—median (IQR)	2.1 (0.7–4.6)	6.3 (2.0–38.1)	6.2 (3.0–152.8)
HCC during FU-n (%)	0	4 (16.7%)	-
HCC at the time of the sample-n (%)	-	-	
Active			19 (82.6%)
No active			4 (17.4%)
N° of nodules—median (IQR)	-	-	2.9 (0–15.0)
Size of nodules (mm)—median (IQR)	-	-	31.8 (12.0–110.0)
Metastasis-n (%)	-	-	1 (4.3%)
BCLC-n (%)			
0			2 (8.7%)
A			10 (43.8%)
B			4 (17.4%)
C			3 (13.0%)
D			-
Previously treated for HCC-n (%)	-	-	
Yes			14 (60.9%)
No			9 (39.1%)
mRECIST *-n (%)	-	-	CR 4 (28.6%) PR 4 (28.6%) SD 2 (14.2%) PD 4 (28.6%)
Deaths-n (%)	0	4 (16.7%)	3 (13.0%)
Median FU (months)—median (IQR)	388.7 (4.0–1428.0)	71.7 (2.0–1493.0)	35.1 (1.0–116.0)
Altered profile-n (%)	0	1 (5.0%)	5 (21.7%)

* Frequencies calculated considering only previously treated patients. Abbreviations: IQR, interquartile range; MASLD, Metabolic dysfunction-associated Steatotic Liver Disease; MetALD, Metabolic dysfunction-associated Alcohol-Related Liver Disease; MELD, Model for End-stage Liver Disease; CSPH, clinically significant portal hypertension; AFP, alpha-fetoprotein; FU, follow-up; CR, complete response; PR, partial response; SB, stable disease; PD, progression disease.

**Table 2:** Oncological characteristics of patients with altered genomic profile at sample collection.

Patient	AFP (ng/mL)	N° of Nodules	Size of Nodules (mm)	Metastasis	BCLC	Dead	OS (Months)	TF (%)	PD
H18	22,700.0	5	21.0	Yes	C	No	26.0	31.0%	Yes
H19	3.0	3	20.0	No	A	No	25.0	14.6%	Yes
H20	470.0	15	15.0	No	C	Yes	9.0	27.9%	Yes
H21	4.6	5	17.0	No	B	Yes	17.0	3.1%	Yes
H22	8.6	6	55.0	No	C	Yes	1.0	32.6%	Yes

Abbreviations: Hn, patient with hepatocellular carcinoma; AFP, alpha-fetoprotein; N°, number; BCLC, Barcelona Clinic Liver Cancer; OS, overall survival; TF, tumor fraction; PD, progressive disease.

**Table 3:** Clinical and oncological characteristics of patients stratified by ctDNA status.

Variable	Overall (n = 23)	Undetectable ctDNA (n = 18)	Detectable ctDNA (n = 5)	*p*
Sex-n (%)				0.133
Males	17 (73.9)	12 (66.7)	5 (100)	
Females	6 (26.1)	6 (43.3)	0 (0)	
Age at the time of the sample—median (IQR)	65 (60–72)	67 (59–76)	61 (52–69)	0.257
Etiology-n (%)				0.461
Alcohol	5 (21.7)	5 (27.8)	0 (0)	
MetALD	2 (8.7)	1 (5.6)	1 (20.0)	
Viral	13 (56.5)	10 (55.6)	3 (60.0)	
Viral + Alcohol	3 (13.0)	2 (11.1)	1 (20.0)	
AFP (ng/mL)—median (IQR)	6.2 (3.0–152.8)	5.6 (2.8–74.9)	8.6 (3.8–11585)	0.359
MELD—median (IQR)	9 (8–12)	9 (8–12)	10 (8–17)	0.403
CHILD-n (%)				0.109
A	19 (82.6)	15 (83.3)	4 (80.0)	
B	3 (13.0)	3 (16.7)	0 (0)	
C	1 (4.3)	0 (0)	1 (20.0)	
CHILD—median (IQR)	6 (5–7)	6 (5–7)	6 (5–9)	0.257
Number of nodules—median (IQR)	2 (1–5)	1 (1–4)	4.7 (1–11)	0.005
Max size—median (IQR)	22.0 (16.5–43)	24.0 (17.0–46.0)	20.0 (16.0–38.0)	0.500
CSPH †-n (%)				0.423
Yes	17 (73.9)	14 (77.8)	3 (60.0)	
No	6 (26.1)	4 (22.2)	2 (40.0)	
Metastasis-n (%)				0.138
Yes	1 (4.3)	0 (0)	1 (20.0)	
No	22 (94.7)	18 (100)	4 (80.0)	
BCLC-n (%)				0.010
0	2 (8.7)	2 (11.1)	0 (0)	
A	10 (43.5)	9 (50.0)	1 (20.0)	
B	4 (17.4)	3 (16.7)	1 (20.0)	
C	3 (13.0)	0 (0)	3 (60.0)	
mRECIST-n (%)				0.001
CR	4 (17.4)	4 (22.2)	0 (0)	
PR	4 (17.4)	4 (22.2)	0 (0)	
SD	2 (8.7)	2 (11.1)	0 (0)	
PD	4 (17.4)	0 (0)	4 (80.0)	
Progression-n (%)				<0.0001
*Yes*	4 (17.4)	0 (0)	4 (80.0)	
*No*	10 (60.9)	10 (55.6)	0 (0)	
Status ‡-n (%)				0.246
Active	4 (17.4)	14 (77.8)	5 (100)	
No active	10 (43.5)	4 (22.2)	0 (0)	
Previously treated-n (%)				0.322
*Yes*	14 (60.9)	10 (55.6)	4 (80.0)	
*No*	9 (39.1)	8 (44.4)	1 (20.0)	
Death-n (%)				<0.0001
Yes	3 (13.0)	0 (0)	3 (60.0)	
No	20 (87.0)	18 (100)	2 (40.0)	

† CSPH diagnosis was based on the presence of unequivocal signs of portal hypertension (splenomegaly, ascites, varices) and platelet count < 100 × 10^9^/L. ‡ Active nodules refer to patients with active HCC at the time of the study, including those who were treatment-naïve and those previously treated but experiencing either recurrence or non-response to therapy.

## Data Availability

The original contributions presented in this study are included in the article/Supplementary Material. Further inquiries can be directed to the corresponding author.

## Supplementary Materials

The following supporting information can be downloaded at: https://www.mdpi.com/article/10.3390/cancers17071115/s1, Figure S1: cfDNA yield in plasma of patients with chronic hepatitis, cirrhosis, and HCC; Figure S2: Representative image of a negative TF sample; Table S1: sWGS results for cirrhotic and HCC patients who tested negative.

## Abbreviations

The following abbreviations are used in this manuscript:

HCC	Hepatocellular carcinoma
AFP	Alpha-fetoprotein
TF	Tumor fraction
ccfDNA	Circulating cell-free DNA
sWGS	Shallow whole genome sequencing
ctDNA	Circulating tumor DNA
SCNAs	Somatic copy number alterations
CNVs	Copy number variations
HBV	Hepatitis B virus
HDV	Hepatitis D virus
HCV	Hepatitis C virus
LB	Liquid biopsy
IQR	Interquartile range
CLD	Chronic liver disease
CSPH	Clinically significant portal hypertension
CR	Complete response
PR	Partial response
SD	Stable disease
PD	Progression disease
