# Supplementary material for: Somatic Copy Number Alterations in Circulating Cell-Free DNA as a Prognostic Biomarker for Hepatocellular Carcinoma: Insights from a Proof-of-Concept Study
Source: Cancers (Basel). 2025 Mar 26;17(7):1115. doi: 10.3390/cancers17071115 (PMC11988118; doi:10.3390/cancers17071115)
Supplement: Supplementary file 1 [file cancers-17-01115-s001.zip › cancers-3516054-supplementary.pdf]

Table S1: sWGS results for cirrhotic and HCC patients who tested negative.

Patient ID	Patient Group	TF (%)
C1	Cirrhosis	0.00
C2	Cirrhosis	0.00
C3	Cirrhosis	0.00
C4	Cirrhosis	0.00
C5	Cirrhosis	0.00
C6	Cirrhosis	0.00
C7	Cirrhosis	0.00
C8	Cirrhosis	0.00
C9	Cirrhosis	0.00
C10	Cirrhosis	0.00
C12	Cirrhosis	0.00
C13	Cirrhosis	0.00
C14	Cirrhosis	0.00
C15	Cirrhosis	0.00
C16	Cirrhosis	0.00
C17	Cirrhosis	0.00
C18	Cirrhosis	0.00
C19	Cirrhosis	0.00
C20	Cirrhosis	0.00
C21	Cirrhosis	0.00
C22	Cirrhosis	0.00
C23	Cirrhosis	0.00
H1	HCC	0.00
H2	HCC	0.00
H3	HCC	0.00
H4	HCC	0.00
H5	HCC	0.00
H6	HCC	0.00
H7	HCC	0.00
H8	HCC	0.00
H9	HCC	0.00
H10	HCC	0.00
H11	HCC	0.00
H12	HCC	0.00
H13	HCC	0.00
H14	HCC	0.00
H15	HCC	0.00
H16	HCC	0.00
H17	HCC	0.00
H23	HCC	0.00

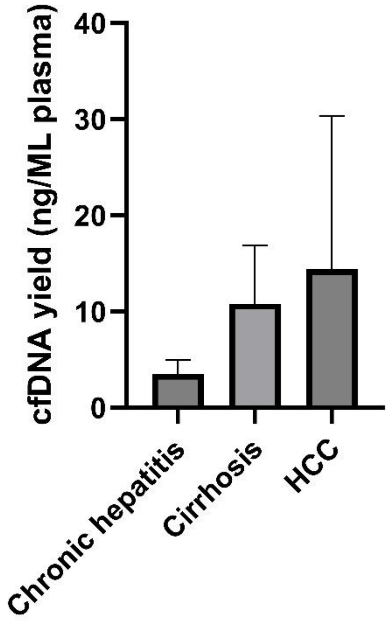

Figure S1: cfDNA yield in plasma of patients with chronic hepatitis, cirrhosis, and HCC

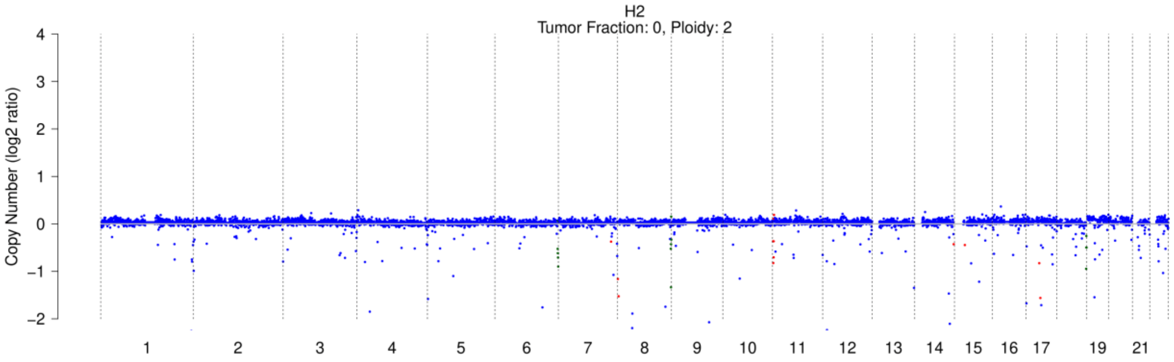

Figure S2: Representative image of a negative TF sample
